# Superficial Siderosis and Sudden Sensorineural Hearing Loss: A Case Report and Review of the Literature

**DOI:** 10.1155/2013/937840

**Published:** 2013-05-22

**Authors:** Kirsti S. V. Lee, Niranjan Sritharan, Allan Forrest

**Affiliations:** Department of Otolaryngology, Head and Neck Surgery, Royal North Shore Hospital, St Leonards, NSW 2065, Australia

## Abstract

This case report highlights an unusual case of sudden sensorineural hearing loss related to superficial siderosis (SS). Our patient had a craniotomy for medulloblastoma 23 years earlier, and this may represent a delayed complication related to this procedure. Magnetic resonance imaging (MRI) remains the key diagnostic investigation to illustrate the imaging features of superficial siderosis and exclude other pathologies. Increased awareness of progressive and sudden hearing complications caused by SS is important in the otolaryngologic community to expedite management and better counsel patients during the consent process.

## 1. Introduction

 Superficial siderosis (SS) classically affects the leptomeninges, brain, brainstem, cerebellum, cranial nerves and spinal cord secondary to hemorrhage and hemosiderin deposition [1]. Hemosiderin, a by-product of iron, releases cytotoxic hydroxyl radicals that cause bilateral sensorineural hearing loss in 95% of patients and cerebella signs [2]. Common etiologies include traumatic nerve root avulsion, bleeding, CNS neoplasm, vascular malformations, and aneurysm.

 It is an uncommon condition with only approximately 100 cases reported [3]. Early clinical suspicion for SS should be raised for patients who present with progressive bilateral hearing loss with the adjunct of MRI for confirmation of diagnosis as it can cause severe long-term neurological effects.

 This case report provides an account of a young male who presented with sudden sensorineural hearing loss.

## 2. Case Presentation

 A 30-year-old Caucasian male patient presented to our clinic with sudden-onset left-sided sensorineural hearing loss and nonpulsatile tinnitus. His medical background was remarkable for a medulloblastoma treated with a suboccipital craniotomy and tumour resection with adjuvant radiotherapy at the age of seven. The patient had no postoperative complications or evidence of tumour recurrence on long-term followup. Examination revealed bilateral sensorineural hearing loss on tuning fork testing with normal otoscopic findings. Dysdiadochokinesia, past pointing, and intention tremor were also noted affecting the patient's left upper limb. Audiometry showed bilateral down-sloping moderate-severe sensorineural hearing loss, which was slightly worse on the left side. No prior audiometry was available for comparison. 

## 3. Discussion

 The pathogenesis of SS results from chronic hemorrhage into the subpial layers with a predilection for the superior vermis, crests of the cerebellar folia, basal frontal lobe, temporal cortex, brainstem, spinal cord, nerve roots, and cranial nerves one and eight. The sources of hemorrhage are cerebrospinal fluid (CSF) cavity lesions, tumors, and vascular abnormalities [4]. 

 The exhaustion of ferritin biosynthesis by the microglial cells as a result of iron overload subsequently causes neuronal injury [4]. Cerebellar involvement is preferential due to the accelerated nature of ferritin synthesis in the Bergmann glia of the cerebellum [5]. Common clinical presentation includes slowly progressive cerebellar ataxia with sensorineural hearing loss being the first symptom with or without tinnitus [6]. The eighth cranial nerve is particularly susceptible to iron deposition due to its long glial composition [4] thus being the likely cause of sensorineural hearing loss in this patient. 

 The patient's history of previous intradural cranial surgery for medulloblastoma is a risk factor for later development of SS [7] and may be related to postsurgical CSF neovascularisation. Surgical management is the treatment of choice to correct any source of active bleeding; however, in this patient, no source was found [8]. The patient was treated with a tapering course of oral prednisone over 14 days, commencing at 1 mg/kg/day. Repeated audiometry performed 3 months after presentation showed stable thresholds. 

**Figure 1:**
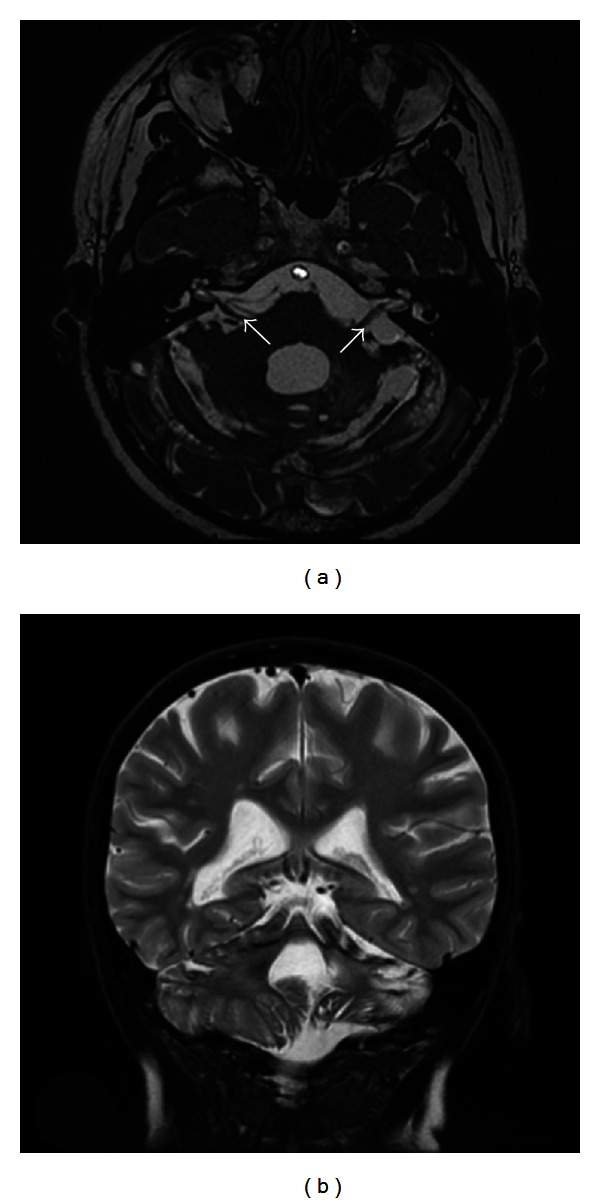
T2-weighted FIESTA magnetic resonance images of superficial siderosis. (a) Axial view of hypointense bands encompassing the vestibular cochlear nerves bilaterally and the cerebellar folia, suggestive of superficial siderosis. (b) Coronal view of cerebella atrophy.

 T2-weighted fast imaging employing steady-state acquisition (FIESTA) magnetic resonance imaging (MRI) revealed hypointense thickened bands along the vestibulocochlear nerves bilaterally which represent classic diagnostic features of SS [1]. FIESTA phase MRI allows for finer sequences to allow more detailed imaging. Imaging also revealed left-sided cerebella atrophy. No eighth nerve tumour or cerebellopontine angle mass was detected (Figure 1).

## 4. Conclusion

 SS is a well-described delayed complication following craniotomy. Patients and their carers must be counseled regarding its association with sensorineural hearing loss, both sudden and progressive, during the consent process. Increased awareness of SS amongst the otolaryngologic community is important so that appropriate audiometric testing, imaging, and management may be expedited.
